# Amyloid single-cell cytotoxicity assays by nanomotion detection

**DOI:** 10.1038/cddiscovery.2017.53

**Published:** 2017-08-21

**Authors:** Francesco S Ruggeri, Anne-Laure Mahul-Mellier, Sandor Kasas, Hilal A Lashuel, Giovanni Longo, Giovanni Dietler

**Affiliations:** 1 Institute of Physics, Ecole Polytechnique Fédérale de Lausanne (EPFL), Lausanne CH-1015, Switzerland; 2 Laboratory of Molecular and Chemical Biology of Neurodegeneration, Brain Mind Institute, Faculty of Life Sciences, École Polytechnique Fédérale de Lausanne (EPFL), Lausanne CH-1015, Switzerland; 3 Istituto di Struttura della Materia, Consiglio Nazionale delle Ricerche, Rome, Italy

## Abstract

Cells are extremely complex systems able to actively modify their metabolism and behavior in response to environmental conditions and stimuli such as pathogenic agents or drugs. The comprehension of these responses is central to understand the molecular bases of human pathologies, including amyloid misfolding diseases. Conventional bulk biological assays are limited by intrinsic cellular heterogeneity in gene, protein and metabolite expression, and can investigate only indirectly cellular reactions in non-physiological conditions. Here we employ a label-free nanomotion sensor to study single neuroblastoma cells exposed to extracellular monomeric and amyloid *α*-synuclein species in real-time and in physiological conditions. Combining this technique with fluorescence microscopy, we demonstrate multispecies cooperative cytotoxic effect of amyloids and aggregate-induced loss of cellular membrane integrity. Notably, the method can study cellular reactions and cytotoxicity an order of magnitude faster, and using 100-fold smaller volume of reagents when compared to conventional bulk analyses. This rapidity and sensitivity will allow testing novel pharmacological approaches to stop or delay a wide range of human diseases.

## Introduction

The complexity of the brain architecture and its cellular heterogeneity are central limitations to evaluate the specific responses, biological functions and causes of cytotoxicity in living organisms when subjected to external stimuli such as the exposition to pathogenic agents or drugs. Conventional biological assays that investigate cellular modifications in bulk conditions are affected by an intrinsic variability, which can be explained by a heterogeneity in the expression levels of genes, proteins or metabolites in the living systems under consideration.^1^ These methodologies can evaluate cellular stress and vitality by using spectroscopic indirect techniques, exploiting fluorescent dyes or absorbance measurements. Thus, by only considering the average cellular reactions from a large population of cells,^2,3^ they might conceal important but subtle individual cellular responses.^4^ Furthermore, these bulk assays can assess mid- and long-term cellular responses generally arising long after the exposure to chemical or physical stimuli. For this reason, these methods could not account for a phenotypic modification in the studied cells during the assessment and they could mask the dynamics of early events that are important in the initiation and long-term propagation of cellular responses to this exposure.

The current lack of understanding of the molecular mechanisms involved in the onset of amyloid-related human diseases, such as Parkinson’s disease (PD),^5–7^ is partially caused by this difficulty in studying the response of populations of cellular systems with intrinsic variability. In PD, the presynaptic protein *α*-synuclein (*α*-syn) accumulates in intracellular fibrillar aggregates, which are recognized as main pathological hallmarks of the disease.^8,9^ Recently, animal and cellular models have shown that *α*-syn can be released extracellularly and taken up by neighboring cells, where it can seed the aggregation of endogenous *α*-syn.^10,11^ Thus, a keen interest has aroused for the potential contribution of extracellular *α*-syn in the neurodegeneration and PD progression.^12–14^ Therefore, there is an urgent need for real-time assays that allow single-cell handling, without interference from neighboring cells, and that are able to overcome the intrinsic limitations of bulk assays.

Towards this goal, we developed a new experimental approach to study single-cell responses using an innovative nanomotion sensor^15^ that can transduce in measurable fluctuations the nanoscale metabolically-related movements of cells.^16^ This methodology allows monitoring in real-time and at a single-cell scale the response to external stimuli in the order of hours, way before the conventional high-throughput bulk assays, providing a platform of unparalleled sensitivity and time resolution to monitor different biological systems, such as bacteria, yeast and mammalian cells with unprecedented resolution.^15–18^ Indeed, previous works have demonstrated how micron-sized nanomechanical sensors can transduce even the very small fluctuations produced by conformational changes in proteins and that the vibrations of just few proteins can produce a measurable fluctuation of the sensor.^19^

Here we applied this nanomotion detector to study the specific responses of a neuron-model system exposed to physiological concentrations of extracellular monomeric and toxic amyloid aggregated species of *α*-syn. We used a dopaminergic human neuroblastoma cell line (M17) to evaluate directly the neurotoxic mechanisms and cellular responses of these cells upon exposure to different *α*-syn species, monomeric or aggregated, in order to understand how these agents might influence cellular metabolism and viability and the role of aggregation in cellular toxicity. As a preliminary step, we studied the cytotoxicity of these species by conventional bulk culture studies. Next, we combined fluorescence microscopy with nanomotion investigations, to monitor in real-time the activity of the cells during the exposure to the *α*-syn species, with a particular focus on the conditions that produce cytotoxicity. Our analyses show that single-cell measurements yield new insights in the cellular response to amyloid species of *α*-syn.

Overall, its peculiarities make this an ideal innovative research platform to test new pharmacological methods to prevent or delay the formation of the toxic species on the aggregation pathway and to block neurodegeneration and the onset of amyloid-related pathologies.

## Results

### Cytotoxicity studies

At first, we performed a complete characterization of the biophysical and structural properties of the recombinant *α*-syn monomeric and aggregated proteins, using bulk and single-molecule techniques: Thioflavin T (ThT), Circular Dichroism (CD) and atomic force microscopy (AFM) imaging (Supplementary Information 1, Supplementary Figure S1).^20^ In our cytotoxicity studies, we considered at the following *α*-syn preparations: (a) freshly filtrated monomers, (b) a solution of mature fibrillar aggregates and (c) a stable mixture of oligomeric and mature fibrillar species, which we called crude mixture.^20–22^ Then, we assessed their toxicity towards human dopaminergic neuroblastoma M17 cells in culture. We exposed the M17 cells to monomeric, fibrillar or to the crude mixture of *α*-syn. After 4 days of treatment, we collected the M17 cells and stained them with Propidium Iodide (PI), a vital dye that enters only in cells with disrupted plasma membrane. The percentage of PI-positive cells (dead cells) was quantified by flow cytometry.^23,24^ As previously shown,^22^ monomeric and fibrillar *α*-syn were not toxic to the M17 cells over the concentration range from 20 nM to 2 *μ*M, whereas the addition of the crude mixture induced significant cell death after 4 days of treatment from concentrations as low as 200 nM (Supplementary Figure S2). The conventional plate-based toxicity analyses agreed well with the single-cells experiments on the non-toxic nature of the monomeric *α*-syn.^22^ It is known that the monomeric, soluble form of *α*-syn is normally present in high concentration at the presynaptic terminals of the brain.^21,25–27^ In these physiological conditions, soluble monomers do not possess a neurotoxic effect. In contrast, most of the available evidences indicate that the aggregation of *α*-syn is essential for the induction of the pathological effects associated with PD.^28–30^

**Figure 1:**
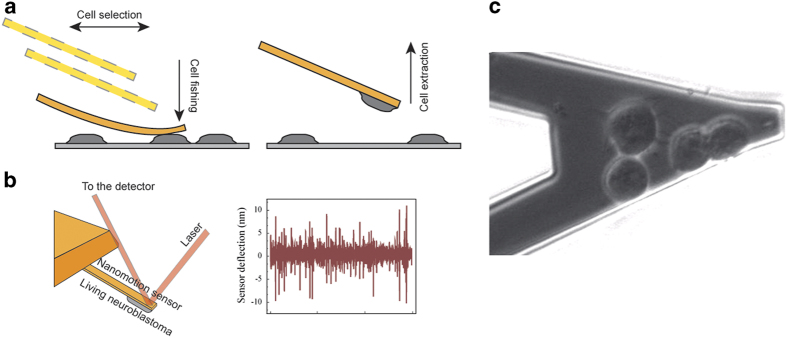
Schematics nanomotion sensor setup. (**a**) The functionalized cantilever is approached to a cell and gently pressed over it. The cell is immobilized on the surface of the sensor and the cantilever is retracted to allow the cell firm adhesion. (**b**) Conventional laser-based detection system of the vertical fluctuations of the sensor and resulting nanomotion signal of the cantilever deflection. (**c**) Optical image of four neuroblastoma cells adhering on the cantilever and linked by their neurites (Supplementary Movies S1–S3).

The subsequent step was the toxicity analyses of the M17 cells exposed to the different *α*-syn species at the single-cell level using the nanomotion assays. We used the micrometric motors of the AFM to attach few living cells (typically 3–5 cells per experiment) to the surface of the sensor (Figure 1a) as previously described.^16^ We collected the deflections of the cantilever as a function of time using a conventional laser-based detection system and we used them to evaluate the activity of the cells (Figure 1b). The movements of the viable neuroblastomas caused an increase of the fluctuations (Supplementary Figure S3 and Supplementary Movie S1), while the inactivation or death of the cells resulted in a reduction of the movements of the sensor.

In order to exploit the peculiarities of the nanomotion sensor, we studied the cellular response of M17 neuroblastomas exposed to *α*-syn monomers, fibrils or to the crude mixture of species using a variety of time-resolved experimental pathways in a wide range of protein concentrations in solution ranging from 0.05 to 120 nM. Notably, in all cases, a single exposure to any of the amyloidogenic species did not induce cytotoxicity (described in details in the Supplementary Information 2, Supplementary Figure S4); this was a peculiarity of the nanomotion experimental setup. In order to investigate the possibility of cooperative effects and secondary processes in inducing cytotoxicity,^29,31^ we performed a two-step *α*-syn injection. A first injection of half of the target concentration was typically followed by a 1 h stabilization period and subsequently the concentration was increased to the target concentration.

**Figure 2:**
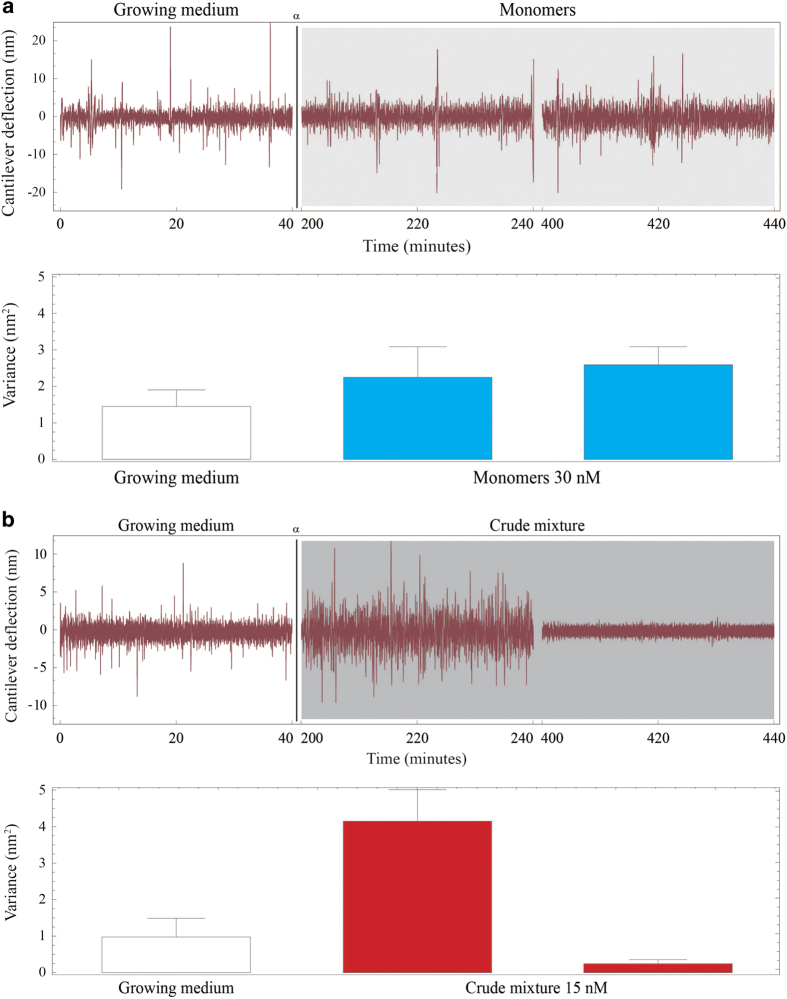
Nanomotion single-cell cytotoxicity studies. Typical nanomotion response of M17 neuroblastoma cells exposed to (**a**) monomeric *α*-syn and (**b**) the crude mixture of oligomers and fibrils. In both cases, the amyloid species were injected in a two-step procedure involving half dose—1 h stabilization—half dose. The A point in the graph is relative to the last injection. After 4 h from the injection of the *α*-syn, the monomers do not cause a toxic reaction while the crude mixture had led the cells to death. More than 15 independent repeats were completed for each case. The histograms depict the average variance and the error bars indicate the variability of the variance over the* *chosen time-step.

In the case of *α*-syn monomers, the cellular viability of the M17 cells was only slightly affected by the exposure to the protein. Indeed, while the first dose had no measurable effect on the cell activity, the nanomotion signal remained constant for >4 h after the injection of the second dose of proteins (that is, >8 h from the start of the experiments, Figure 2a). Comparing the experiments involving a single-monomer injection to the results after the two-step procedure, we measured a 30–50% increase in the variance of the fluctuations after the second exposure, probably indicating a non-fatal cellular reaction to the proteins (Figure 2a and Supplementary Figure S4a). This response could be detected only because of the sensitivity of the nanomotion sensor, since the concurrent optical investigation did not show any variation in the cell motility (Supplementary Movie S2). From the toxicity point of view, the cells were still alive and moving after 8 h from the start of the measurements, as in the control experiments (Supplementary Figure S3), indicating that the monomers did not induce cytotoxic response in the M17 cells. Similarly, after several hours from the injection, the purified fibrillar *α*-syn samples did not induce any toxicity in the M17 cells, which were still active, both in the case of single injection (Supplementary Figure S4c) and when employing a double-injection protocol (Supplementary Figure S5). Next, we investigated the response of the neuroblastomas cells to the *α*-syn crude mixture delivered, as before, in two-step injection protocols with a 1 h stabilization period. The injection of the first half of the proteins did not modify the activity of the cells, which after 1 h were still exhibiting an activity comparable to the control single-step experiments (Supplementary Figure S4b). Remarkably, as depicted in Figure 2b, within the first 30 min from the second injection of the crude mixture, the cells increased their net fluctuations (the variance of the fluctuations increased up to 4-fold), indicating a strong cellular response to the crude *α*-syn mixture. The increase in fluctuations lasted in average about 2 h and ended abruptly with the cells undergoing cell death, as evidenced by the flatness of the nanomotion pattern, which indicated the absence of any cell-related activity. Moreover, the optical images showed that the cells increased their activity before swelling and, in many cases, exploding (Supplementary Movie S3). Overall, the addition of *α*-syn crude mixture in one single dose had no measurable effect on the M17 cells isolated on the cantilevers, while the two-step sequential injection induced a significant toxicity and induced a rapid cell death. Most of the cellular responses were undetectable using the conventional optical analyses, in which the M17 cells appeared only to abruptly reduce their microscopic movements and die, often after some osmotic swelling.

The real-time monitoring and high sensitivity of the nanomotion sensor was fundamental to reveal the complex dynamic responses of the cells. Furthermore, in order to characterize fully the cytotoxic effect of the crude mixture as a function of the injected proteins concentration, we performed a series of nanomotion, dose-dependence experiments. These results show that even a small concentration of the mixture such as 2 nM is capable to produce a cytotoxic effect on M17 cells isolated on a cantilever. (Supplementary Figure S6).

Finally, to allow a good comparison between these population-level experiments to the subsequent nanomotion sensor assays, we treated the cells in the wells with a two-step injection of monomers and crude mixture. At first, we injected half of the final chosen concentration of the *α*-syn and delayed the injection of the second dose of proteins for 1 or 24 h. The conventional plate-based toxicity analyses, both at single exposure and at two-step injection, agreed well with the single-cell experiments, confirming the cytotoxic nature of the aggregated mixture. This is in good agreement with previous results by our group^22^ and by other researchers,^10,32–35^ suggesting that the process of aggregation and fibril formation play central roles in amyloid toxicity (Supplementary Figure S7).^36^

The comparison between our experiments in multi-well plates and at the single-cell level highlighted the importance of the timing of the *α*-syn delivery in the determination of the cellular demise. The experiments on cantilevers required a two-step injection of *α*-syn crude mixture to produce cell death, while for the multi-well-based analyses, a single injection was sufficient to induce cytotoxicity. This is probably related to the experimental geometry: in the nanomotion setup, the insoluble material sediments quickly and is not continuously in contact with the cells throughout the incubation. Since the timescale for the multi-wells experiments can be days, this sedimentation can lead to the formation of new amyloidogenic species, with unforeseen interactions with the cells. Notably, to deliver information on cellular response and measure cellular cytotoxicity, the nanomotion experiments required an experimental time (4–12 h) and a minimal crude mixture concentration (2 nM) up to two orders of magnitude smaller than the conventional plate analyses we performed (4 days, 200 nM). Also, this high sensitivity of the M17 cells in the nanomotion sensor assays can be tentatively attributed to the experimental geometry. The small number of cells on the sensor (at most 5 cells were present on the cantilever at the same time) and the space constraints imposed by the cantilever do not favor intercellular interactions and cell to cell communications, making the individual cells certainly more sensitive to their environment.^37–39^

### Cooperative cytotoxicity of amyloids species

**Figure 3:**
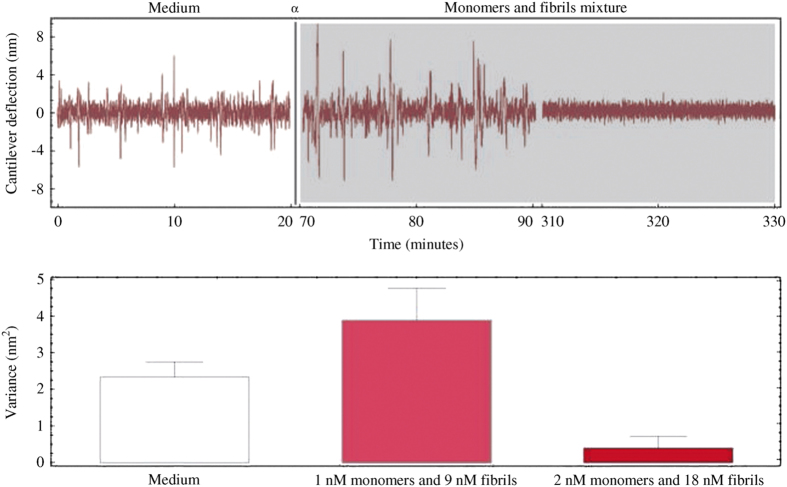
Nanomotion experiments using a mixture of *α*-syn monomers and fibrils. Using a two-step procedure, the M17 cells undergo a rapid cytotoxic reaction and, after <4 h, appear to have died. The histograms depict the average variance and the error bars indicate the variability of the variance over the chosen time-step.

To understand better the cooperative role of monomeric and fibrillar species in the onset of cytotoxicity and to investigate the role of a two-step injection for the nanomotion experiments, we exposed the M17 cells either to a mixture of monomeric and fibrillar species (Figure 3) or to a sequential exposure to the two species in different order (Supplementary Table S1). In these experiments, we maintained constant the final concentration of the *α*-syn species at 20 nM. The monomers and fibrils mixture caused cytotoxicity in the M17 cells following a pattern very similar to the crude mixture case. A single exposure did not produce cell death, while a two-step exposure always led to cytotoxic response. On the other hand, the sequential injection of monomeric and fibrillar species always led to cell death and the relative concentration of monomers and fibrils had very little influence on the outcome. (Supplementary Table S1) This supports our results involving the crude mixture, since monomeric and fibrillar solutions always contain a certain amount of oligomeric species. Remarkably, the speed of the cell death depended on the sequence of exposure to each specific *α*-syn species. In fact, when the cells were exposed first to monomers and next to the fibrils, we observed a timing of the cytotoxic effect that was delayed compared to when fibrils where injected first (Supplementary Table S2). This finding support our previous data showing that *α*-syn fibrils that bind to the extracellular cell plasma membrane can serve as anchoring and nucleation points for the aggregation of extracellular monomeric *α*-syn at the cell plasma membrane, which disrupts the membrane permeability leading to cell death.^22,29^

**Figure 4:**
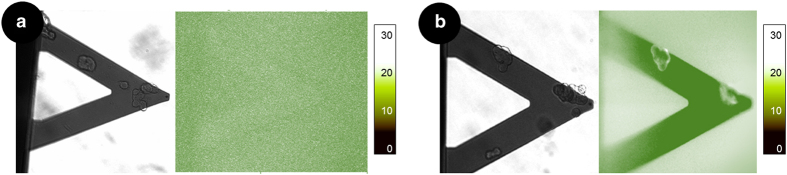
Localization of *α*-syn species. Optical and fluorescence images of M17 cells on a cantilever sensor exposed for 2 h to OG-tagged *α*-syn monomeric (**a**) and fibrillar (**b**) forms. The fibrils are clearly localized on the cell surface while the monomers do not appear to localize on the cells. (The nanomotion response is shown in Supplementary Figure S6).

Finally, to gain further insight into this phenomenon, and in particular to determine the localization of the interaction, we exposed the M17 neuroblastomas to monomeric or fibrillar forms, which were previously tagged with Oregon Green fluorescent dye. For these experiments, we combined the nanomotion sensor with a fluorescence microscope to study simultaneously the viability and activity of the M17 cells and the localization of the highly homogeneous species *α*-syn proteins. As shown in Figure 4a, when exposed to the fluorescent monomeric form, the images do not suggest any specific protein localization on the M17 cells. On the other hand, when the cells were exposed to the fluorescent fibrils, we could observe enhanced fluorescence localized on the cell membrane, thus indicating that fibrillar *α*-syn has bonded on the surface of the M17 cells (Figure 4b). These results support the hypothesis of the cooperative interaction between multiple species of *α*-syn and M17 cells and especially the requirement for two-step *α*-syn injection in our configuration to induce the toxicity in M17 cells.^32,40^ Initially, the insoluble fibrillar species localize on the cell membrane and can act as seeds for the biological action of the soluble monomers and early oligomeric aggregates.^21^

### Membrane permeabilization related cytotoxicity

**Figure 5:**
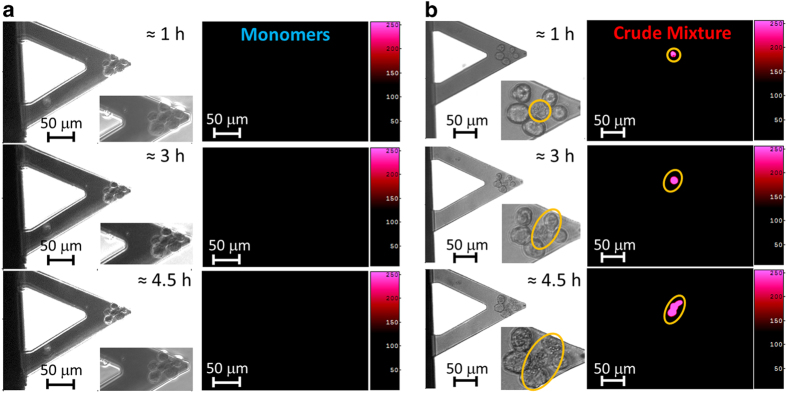
Internalization of the Ethidium homodimer III. Optical and fluorescence images of M17 cells in ETH III-rich medium after 1, 3 and 4.5 h of incubation of cells with (**a**) *α*-syn monomers and (**b**) aggregated *α*-syn crude mixture at 0.12 *μ*M.

It has been suggested that the *α*-syn localization could affect the membrane permeabilization, thus inducing the cytotoxic action. In order to validate this hypothesis, we combined conventional fluorescence and nanoscale techniques to investigate the cellular response to extracellular *α*-syn at the single-cell level. We collected several cells on the cantilever sensor and exposed them to a buffer containing ethidium homodimer III (EtH III), a vital dye whose uptake is a marker of cell death and membrane integrity. After a first incubation period, we treated the cells either with *α*-syn monomers or with the crude mixture. The M17 cells exposed to *α*-syn monomers did not internalize any EtH III, even at the highest concentration of the proteins (120 nM), whereas the exposure to the crude mixture led to a rapid and high internalization of EtH III, even while the cells were still viable. In fact, the time-resolved images showed that the level of the fluorescent dye accumulated over time in the M17 cells (Figure 5). Remarkably, the dye uptake corresponded very well with the increase of the cellular nanomotion, typical of the exposure to the crude mixture before the final cell death (Supplementary Figure S8).

These experiments support our hypothesis that membrane permeabilization is a key contributor to *α*-syn extracellular toxicity. The addition of the extracellular *α*-syn crude mixture, in the range between 2 and 120 nM promotes the loss of plasma membrane integrity and the activation of a strong cellular response and final cytotoxicity, highlighted by the nanomotion experiments,. On the contrary, the monomers do not cause any cell permeabilization, well in agreement with the absence of any cytotoxicity. These results are in agreement with the assumption that the exposure to fibrillar forms or the process of *α*-syn fibrillization play central roles in *α*-syn-mediated neruoblastoma death via membrane permeabilization.^22,23^

## Discussion

Investigating the response of mammalian cells to external chemical or physical stimuli is of paramount importance to study the cellular biology and the origin of human diseases, including neurodegenerative disorders such as Alzheimer’s and Parkinson’s diseases. In the present work, we employed a nanomotion detector to study at the single-cell level, the toxicity of monomeric and aggregated *α*-syn on M17 neuroblastoma cell lines. This phenotypic platform delivers cytotoxic responses very rapidly, almost two orders of magnitude faster than conventional plate-based bulk toxicity assays and at near physiological concentrations, and has the ability to perform label-free analyses, making it the ideal complement to the well-known and established biological techniques at the population level. Furthermore, the nanomotion sensor can perform analyses on single or few cells, and requires a very small analysis chamber. The consequent use of much smaller volumes of buffers, proteins and reagents when compared to conventional bulk techniques, is of paramount importance when characterizing the effects of expensive drugs or chemicals. The comparison between nanomotion and conventional plate assays indicate that the nanomotion single-cell analyses have several advantages over conventional biological experiments, including faster screenings, real-time analyses of the cellular dynamics and time-resolved investigations.

Here, through a careful characterization of the proteins, we were able to demonstrate that the extracellular exposure of cells to a homogeneous monomeric or fibrillar solution of *α*-syn is not toxic, even at a single-cell level. On the other hand, we demonstrated that the cooperative action of the individually non-toxic species led to cytotoxicity and cell death, as in the case of a crude mixture containing residual monomeric and mainly oligomeric and fibrillar species. These results were in good agreement with the analyses at the population level using plate-based toxicity assays.^22^ Furthermore, by combining the nanomotion information with fluorescence microscopy, we investigated the mechanism leading to cytotoxicity, showing that protein-induced loss of membrane integrity ultimately led cells to death. These results highlight the great potential of the nanomotion studies as a new fast and reliable analysis tool to study the molecular basis of amyloid toxicity in misfolding diseases at the single-cell level and, overall, to study a wide range of diseases and biological problems. This could pave the way to a new paradigm in the investigation of toxicity in cells and can become a tool to perform testing of protective drugs, to stop or delay the neurodegenerative toxicity mechanisms.

## Materials and methods

### Substrates, enzymes and reagents

All chemicals, phosphate buffered saline (PBS, pH 7.4), poly-l-lysine, Trypsin-EDTA, DMEM, glycine, F12 medium, fetal bovine serum (FBS), glutaraldehyde, all with analytical grade, were supplied by Life Technologies, (Carlsbad, CA, USA). The uranyl-formate solution was acquired from Electron Microscopy Sciences (Luzern, Switzerland), Ethidium Homodimer III (ETH III) from Promokine (Heidelberg, Germany) and Propidium Iodide (PI) from Millipore (Schaffhausen, Switzerland).

### Preparation and characterization of *α*-syn recombinant proteins: expression and purification of monomers

Human wild-type (WT) *α*-syn was subcloned in pT7-7 plasmid and transferred into the expression strain *E.coli* BL21. After its bacterial expression, *α*-syn was first purified by anion exchange chromatography (AEC) and size exclusion chromatography (SEC) as described in detail by Fauvet *et al.*^41^ and then by reverse-phase HPLC (Waters 600 system) using a Cosmosil C4 preparative column (20×250 mm, 38048) with a linear gradient of 20–70% B (solvent A: H_2_O/0.1% formic acid, solvent B: acetonitrile/0.1% formic acid). The *α*-syn elution was monitored by UV absorbance at 214 and 280 nm and the mass was confirmed by MALDI-TOF-MS (Matrix-assisted laser desorption/ionization-Time of Flight), ESI-MS analysis (Electrospray ionization, Thermo Scientific, Carlsbad, CA, USA) and SDS-PAGE (Sodium dodecyl sulfate-Polyacrylamide gel electrophoresis). Acetonitrile was removed from the *α*-syn containing fractions using a rotary evaporator and the *α*-syn fractions were lyophilized and stored at −20 °C until further use.^22^ To ensure that the preparation of monomeric *α*-syn was free of preformed aggregates or oligomeric forms, as low as dimers, the stock solution was systematically filtered through a 100 kDa filter.

### Preparation and characterization of *α*-syn recombinant proteins: preparation of crude mixture

The *α*-syn crude mixture was generated incubating 800 *μ*l of a 45 *μ*M filtered (100 kDa filter) (Millipore) monomeric *α*-syn solution (50 mM Tris, 150 mM NaCl, pH 7.5) under constant orbital agitation (400 rpm) at 37 °C for 4 weeks. It is of particular importance to highlight that we chose not to sonicate our solutions, since this is known to lead to amyloid fibril breakage and this is suggested as a possible cause of enhanced toxicity.^22^

### Neuroblastoma cell culture

The dopaminergic human neuroblastoma M17 cell line were cultured in humidified air under 5% CO_2_ at 37 °C in Dulbecco’s Modified Eagle Medium and 50% F-12 medium supplemented with 10% of inactivated FBS and 1% of Penicillin/Streptomycin solution. The M17 cells were detached from culture flask using Trypsin-EDTA for 5 min at 37 °C, before further use.

### The nanomotion experiments

The nanomotion analyses were carried out using a Nanowizard III atomic force microscope (JPK, Berlin, Germany), using commercial silicon nitride, micro-cantilevers, with a nominal spring constant of 0.12 N/m (DNP-10 Bruker). The microscope was equipped with a custom liquid cell^42^ and coupled with an Axiovert X optical microscope (Zeiss Microscopy, Germany). All the optical images were collected using a standard 40x objective in the phase-contrast modality through a Progres MFCool digital camera (Jenoptik, Germany). The time-dependent fluctuations of the sensor, linked to the metabolic activity of the biological specimens, were recorded through the JPK control software and we performed the data analysis using a custom software written in LabView (National Instruments, USA). The cantilever fluctuations were recorded with a sampling frequency of 10 kHz.

### Temperature control

The temperature of the analysis chamber was controlled using a custom petri-cell heater, which was calibrated to ensure the cells were kept at 37 °C throughout the entire experiment. Before inserting the cells in the analysis chamber, the system was first stabilized; less than 30 min were needed to obtain a perfectly stable cantilever. All the solutions were left to thermalize for minimally 1 h prior to injection in the analysis chamber. The temperature of all the injected media was controlled just before the injection using a bimetallic temperature sensor (DT120, Rüeger, Switzerland). This ensured that the temperature throughout the entire experimental analysis was constant within 0.1 °C.

### Characterization of the nanomotion sensor

Each cantilever was preliminarily characterized in the fluid environment using the thermal fluctuation method^43,44^ in order to calculate its resonance frequency and the effective spring constant (which were in good agreement with the nominal values). For each experiment, the thermal fluctuation analysis was performed before and after the attachment of the cells, in order to determine any variations of the mechanical properties of the sensor throughout each experiment.

### Functionalization protocol and cell fishing

In our previous works, we have highlighted how the choice of the correct functionalization of the sensor surface is fundamental to obtain the best immobilization efficiency.^16^ To ensure a good attachment of the neuroblastoma cells on the cantilever the sensor was covered with a droplet of 10% (vol/vol) poly-L-lysine and left to incubate for 30 min at room temperature. Next, the sensor was rinsed thoroughly using ultrapure water and transferred it to the analysis chamber, which was flushed with a cellular nourishing buffer containing a small concentration of live cells. Then, we used the AFM coarse and fine movement capabilities to collect some cells,^45^ attaching them near the apical region of the cantilever (typically, 3–5 individual cells were attached per experiment). Once the cells were firmly attached to the sensor, the tip was retracted ~100 *μ*m from the surface and the nanomotion experiments were carried out.

### The nanomotion detector

The setup of the nanomotion detection experiments is described in detail in our pioneering works^15,16^ and is depicted in Figure 1. Each nanomotion experiment started with 1 h stabilization period, which was necessary to verify the viability and the complete adhesion of the cells on the sensor. Throughout this first period, the sensor was immersed in growing buffer and we monitored its fluctuations as a function of time, while recording every 20 s an optical image of the cells. The fluctuations of the sensor were used to investigate the nanometer-scale movements of the adhering cells, while the optical images showed their micron-sized evolution on the cantilever surface. These measurements reflected the basal metabolic activity of the cells. While collecting the nanomotion signal, we recorded time-lapse videos of the cells on the sensor, to check their attachment, their healthiness and to monitor at the micrometer-scale the individual cell movements during the time of the experiment.

After the first stabilization period, we exploited the input-output tubes of the analysis chamber to introduce an enriched medium containing the *α*-syn monomers or crude mixture. We performed several experiments with monomer, fibrils crude mixture protein concentration ranging between 0.05 and 120 nM. The stability of the liquid in the analysis chamber is crucial in our experimental setup. Therefore, after injecting the medium, we waited at least 5 min to ensure that the liquid had stabilized before starting the measurement. The injection of new media in the analysis chamber was performed at very slow rates (<8 *μ*l/s), always verifying through the optical images that the samples on the cantilever sensor were unaffected by the flow. Thus, we were capable of monitoring the movements of the cells and their viability for more than 12 h, to identify the cellular response to the *α*-syn treatment.

To evaluate all the experimental results, we analyzed statistically the fluctuations by calculating their variance and we repeated each experiment at least 3 times to ensure a good repeatability of the results.

### Investigation of cell death by dye exclusion method in M17 cells on the nanomotion sensor

The M17 cells were picked up on the cantilever and, after the stabilization period, they were incubated in the growing medium added with 1 *μ*M of Ethidium Homodimer III (EtH III), a membrane impermeant dye, which enters only in cells with damaged plasma membranes.

To monitor the capacity of the M17 cells to internalize the vital dye, we used a Zeiss Axiovert Z1 system to acquire optical and fluorescence images of the cells on the cantilever. After 15 min of incubation, we collected the baseline images of the neuroblastoma cells (time-point 0). Next, we added the extracellular monomeric *α*-syn, or *α*-syn crude mixture (at 120 nM in both cases) to the cells. The internalization of the EtH III into the cells was recorded by collecting, every 10 min, optical and fluorescence images of the cells for up to 5 h. Each image was acquired with an exposition time of 2 s. In parallel, we monitored the cellular response in terms of nanomotion, in order to understand if the presence of the fluorescent dye had influenced the cells. All the optical images were analyzed by means of SPIP software (Image Metrology, Hørsholm, Denmark).

### Quantification of cell death by dye exclusion method in M17 cells by flow cytometry

The M17 cell lines were plated in 24 wells plates. After 24 h, they were treated with extracellular monomeric *α*-syn, or *α*-syn crude mixture or Tris buffer (50 mM Tris, 150 mM NaCl, pH 7.5) as negative control. Cell death was quantified by the vital dye exclusion method using Propidium Iodide (PI), a vital dye that enters only in cells with compromised membranes. Briefly, the supernatant and the adherent cells were harvested and collected in FACS tube. After 5 min of centrifugation at 500 g, pelleted cells were resuspended in PBS and PI was added to the cells at a final concentration of 2 *μ*g/ml. Cells were then analyzed by flow cytometry using Accuri C6 (BD Biosciences, Allschwil, Switzerland) and FlowJo software (Treestar, Ashland, OR, USA).

### Circular dichroism

Samples were analyzed at room temperature (RT) using a Jasco J-815 CD spectrometer. An average of 10 spectra was collected in the range of 190−250 nm using a 1.0-mm-optical-pathlength quartz cuvette. The data points were acquired every 0.2 nm in the continuous scanning mode at a speed of 50 nm/min with a digital integration time of 2 s and a bandwidth of 1 nm. The processed spectra were obtained by subtracting the baseline signal due to the water and cell contribution from the protein spectra.

### Thioflavin T

Thioflavin T (ThT) fluorescence reading was measured for *α*-syn at 45 *μ*M, diluted at 1.5 *μ*M with a ThT concentration of 10 *μ*M, in a 70 *μ*l solution with pH 8.5 buffer containing 50 mM glycine. A Bucher Analyst AD plate reader was used to measure ThT fluorescence at an excitation wavelength of 450 nm and an emission wavelength of 485 nm. Aliquots taken at different time points were measured in triplicate.

### Electron microscopy analyses

A 10 *μ*l droplet of the sample was deposited on Formvar/carbon-coated 200-mesh copper grids (Electron Microscopy Sciences). The grids were washed twice with 5 *μ*l of ultrapure water and then stained twice with 5 *μ*l of an aqueous 2% w/v uranyl-formate solution and then vacuum-dried from the edges of the grids. Samples were imaged using a Tecnai Spirit BioTWIN electron microscope (TEM) operated at 80 kV with a LaB_6_ source.

### Atomic force microscopy imaging

We characterized the morphology of the different *α*-syn states by Atomic Force Microscopy (AFM). We performed these investigations on positively functionalized mica. After the cleaving, the mica substrate, was incubated with a 10 *μ*l drop of 0.05%(v/v) APTES in Milli-Q water for 1 min at ambient temperature, rinsed with Milli-Q water and then dried by the passage of a gentle flow of gaseous nitrogen. AFM samples preparation was realized at room temperature by deposition of a 10 *μ*l aliquot of full concentrated solution on the surface for 5 min. AFM images were realized in ambient condition by means of a Park NX10 (Park, USA) operating in true non-contact mode and equipped with a silicon tip (Nanosensor, PPP-NCHR, 40 Nm^−1^) with a nominal radius of 7 nm. Image flattening was applied to each image and the analysis was performed using SPIP software.

### Statistical analysis

For the flow cytometry experiments to determine cell death, we performed a one-way ANOVA test followed by a Tukey-Kramer post *hoc* test. The data were regarded as statistically significant at *P*<0.05 based on the Tukey-Kramer post *hoc* test.

Regarding the single-cell analyses, each nanomotion experiment described in this work was the result of a series of least three independent experiments. The representative nanomotion graphs and variance measurements presented in the manuscript are representative of the general behavior of triplicates, which showed all the same behavior. In all graphs, we presented the data with the relative standard deviation. The times-to-death graphs were averaged over minimally three independent experiments.

### Data and materials availability

All the data are freely available on request from the authors.

## Publisher’s note

Springer Nature remains neutral with regard to jurisdictional claims in published maps and institutional affiliations.
