# Supplementary material for: Amyloid single-cell cytotoxicity assays by nanomotion detection
Source: Cell Death Discov. 2017 Aug 21;3:17053–. doi: 10.1038/cddiscovery.2017.53 (PMC5564330; doi:10.1038/cddiscovery.2017.53)
Supplement: Supplementary Information [file cddiscovery201753-s1.docx]

**Supplementary Information**

**Index:**

Supplementary Information 1: Characterization of monomeric and aggregated forms of α-syn

Supplementary Information 2. Nanomotion control experiments

Figures S1-S8

Tables S1-S2

Movies S1-S3

Additional References

**Supplementary Information 1. Characterization of monomeric and aggregated forms of α-syn**

The weak characterization of the protein aggregates is one of the major factors of the discrepancies in the studies involving the cell cytotoxicity of amyloids. Thus, as a preliminary step, we have performed a combination of bulk and single molecule characterization of the α-syn species, which is of fundamental importance for the investigation of the role of amyloids in the disease onset. We carried out full characterization of monomeric α-syn and its aggregated forms in vitro using an array of biophysical methods (**Figure S1**).

At first, we performed a complete characterization of the biophysical and structural properties of the recombinant α-syn preparations.[^1^](#_ENREF_1) For this analysis of paramount importance in the understanding of their interaction with the cells, we used several bulk and single molecule techniques: Thioflavin T (ThT), Circular Dichroism (CD), Electron Microscopy (EM) and Atomic Force Microscopy (AFM) imaging. The AFM analyses showed that the monomeric solution was highly homogeneous contained only a residual amount of small spheroidal early oligomeric species (<5%) with height of ~1 nm (**Figure S1a**).[^2^](#_ENREF_2) After 4 weeks’ incubation at 37°C, the resulting aggregated solution presented the typical fingerprint of amyloids: aggregated oligomers, with larger height (1-9 nm) than the early spheroidal forms, and mature fibrillar species with height of approximately 6-9 nm, which we named crude mixture (**Figure S1b**). EM confirmed the presence of fibrillar aggregates in the incubated species (**Figure S1c**). Consistently, the samples before incubation did not have ThT fluorescence while, after 4 weeks of incubation, the ThT fluorescence was notably increased (**Figure 1d**). This indicated the massive formation of amyloid aggregates. The CD spectrum showed a negative minimum at approximately 222 nm, confirming that, after 4 weeks under shaking condition, the α-syn had formed amyloid β-sheet structure. On the other hand, the same analyses on the filtered α-syn monomeric solution evidenced a spectral signature with a strong negative minimum at approximately 203 nm, typical of proteins in a random coil conformation (**Figure 1e**). In fact, all these results confirmed the purity of the monomeric solution and indicate that the α-syn solution incubated for 4 weeks under shaking conditions is, in fact, a crude mixture containing oligomeric and fibrillar aggregates. Finally, we generated highly fibrillar samples, following previously described protocols, to obtain a homogeneous mixture of mature fibrillar structures.[^3^](#_ENREF_3)


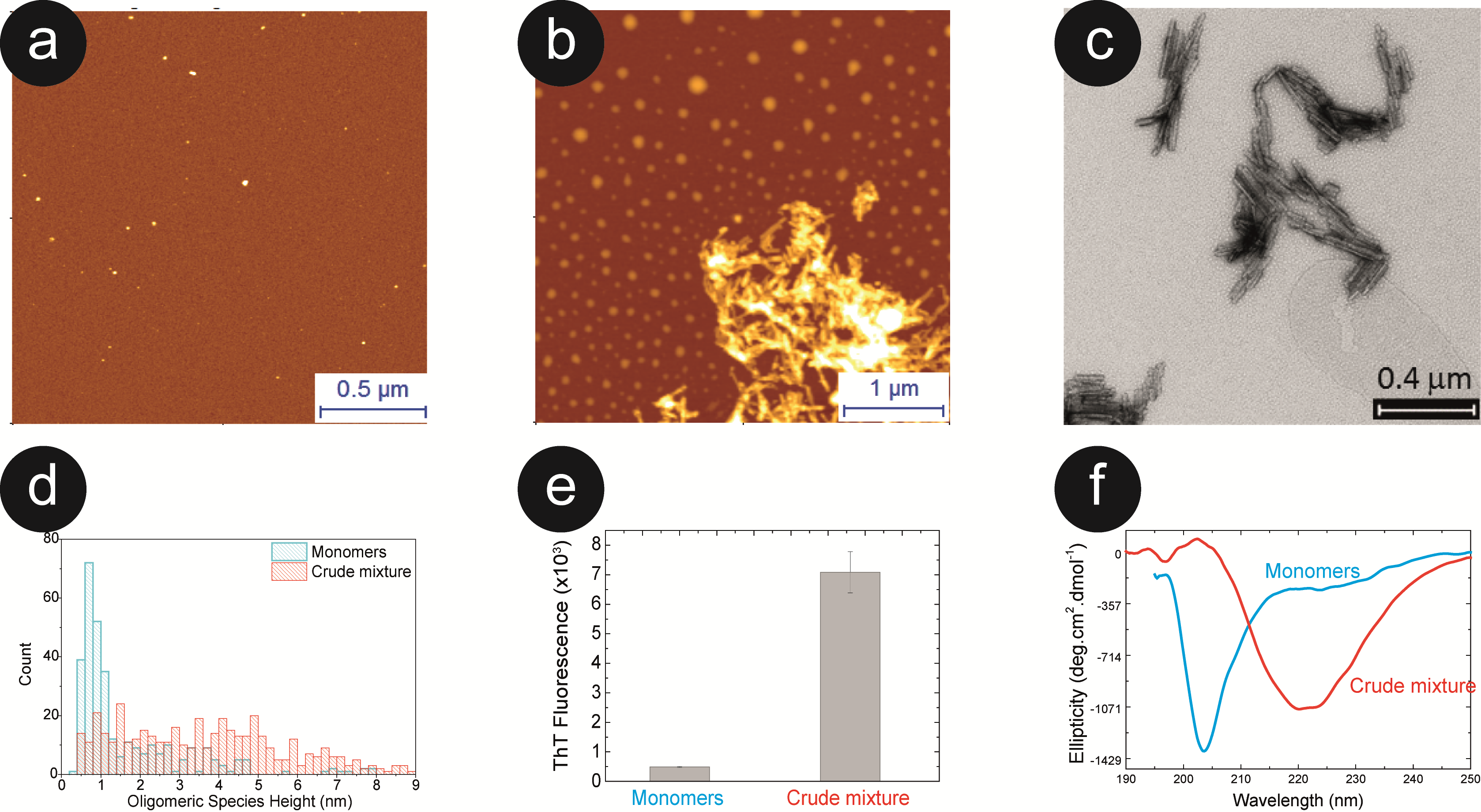


**Figure S1 Amyloid aggregates characterization.** a) AFM image of α-syn before incubation. b) AFM image of α-syn aggregates after 4 weeks of incubation. c) EM of the amyloid aggregates after 4 weeks of incubation. d-e) Thioflavin T (ThT) fluorescence and CD signal of sample before incubation (monomers) and after 4 weeks of incubation.


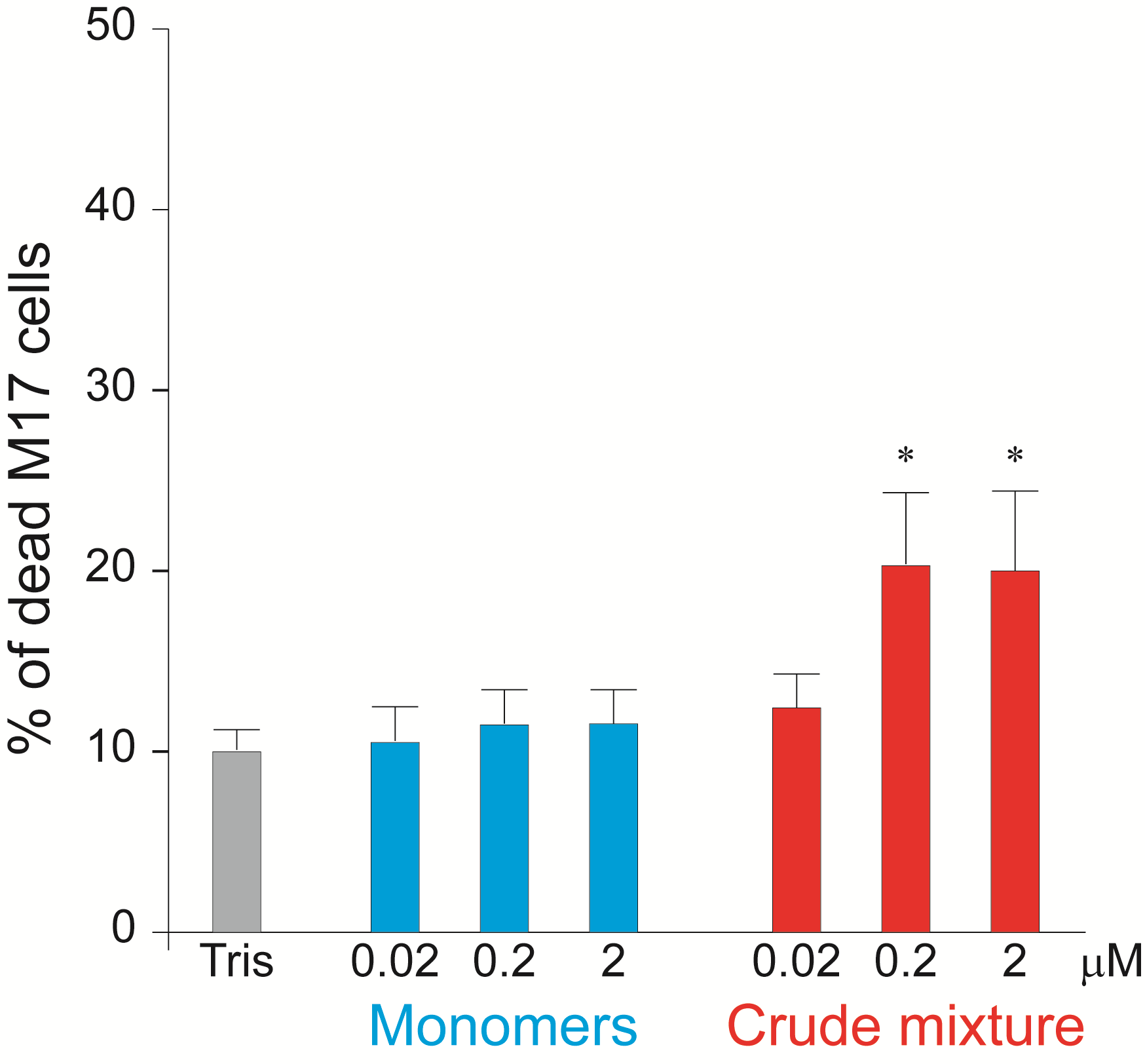


**Figure S2 Cells death quantification by flow cytometry.** The human neuroblastoma M17 cells were treated with Tris buffer (50 mM Tris pH 7.5, 150 mM NaCl; negative control) or α-syn monomers or α-syn crude mixture with the indicated concentrations. After 4 days, the cells were harvested and stained with PI. The percentage of cell death was measured by flow cytometry. Cell death level is expressed as the percentage of cells with loss of plasma membrane integrity (PI positive cells) to the total cell number analyzed by FACS. Data shown represent the mean of three independent experiments performed in triplicate (bars are mean ± S.D.). One-way ANOVA test followed by a Tukey-Kramer post-hoc test were performed (Tris versus α-syn treated conditions), *p<0.01, (b).

**Supplementary Information 2. Nanomotion control experiments**

At first, we monitored over-time the cellular viability and behaviour of the M17 cells attached on the sensor surface and grown in their culture media (control cells). We showed that for more than 8 hours (typically up to 12 hours), in 10 independent experiments, the nanomotion activity of the cells was uniform and the cells were well alive and fully attached to the surface (**Figure S3, Movie 1**). These control experiments indicated that the nanomotion experimental setup allows a controlled analysis of the cell’s metabolic activity for several hours and that its results can be compared directly with the conventional biological assays. Next, we performed several experiments in which, after a first stabilization period of one hour, we injected in the analysis chamber an enriched medium containing either α-syn monomers or the crude mixture. We targeted our injections to reach a final α-syn concentration in the chamber in a range of 0.05 nM to 120 nM. In the first experiments, we performed the injection in one-step, directly to the target concentration. In these cases, throughout the typical 8-12 hour period of each experiment, the cells on the surface of the sensor did not exhibit any kind of measurable reaction to the monomers or the crude mixture, and the fluctuation amplitude remained approximately unchanged. (**Figure S4**)


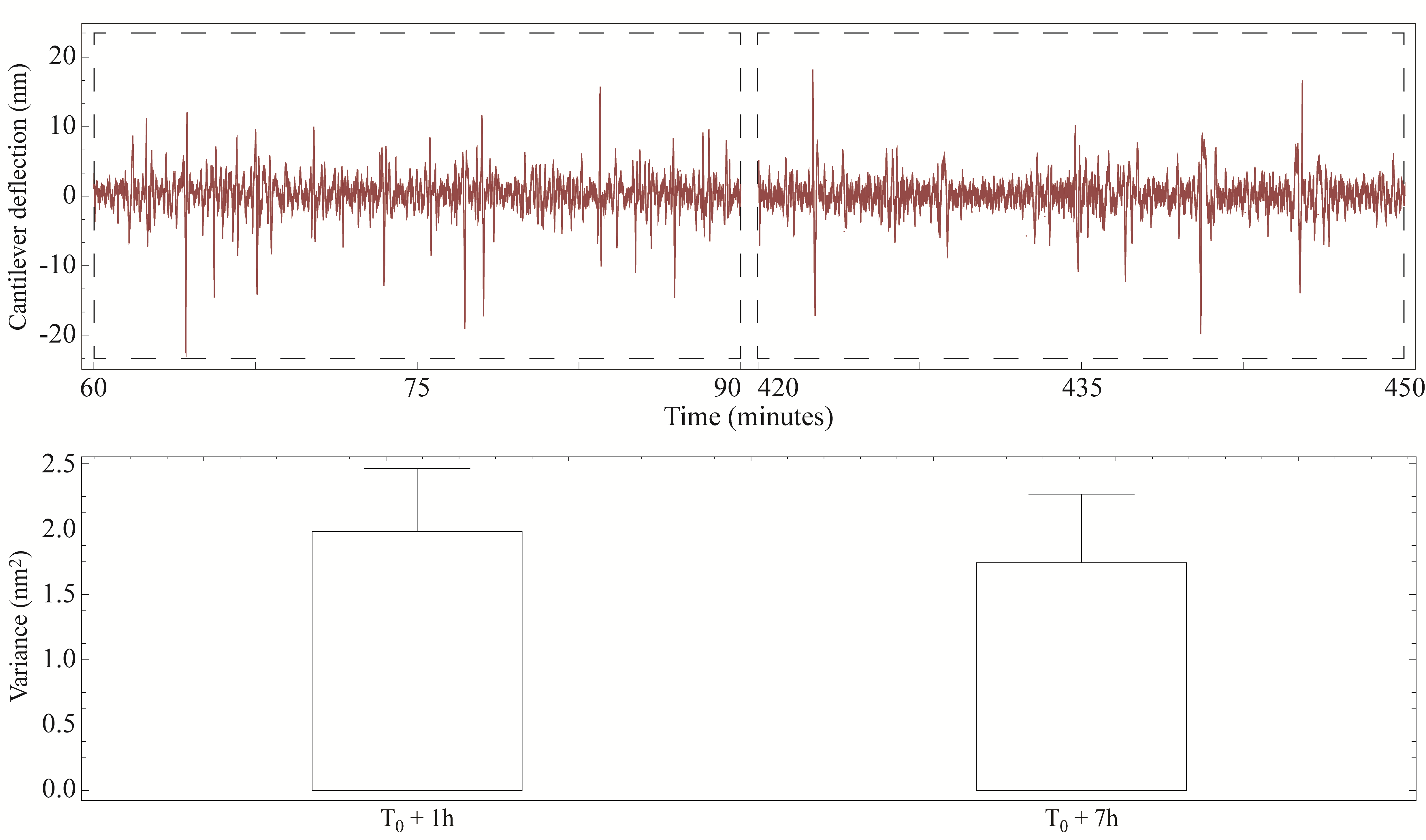


**Figure S3. Monitoring neuroblastomas cell movement.** a) Typical outline of the cantilever deflection arising from a cell in cell culture media (negative control). Overall, 10 independent control experiments were carried out. b) Average variance of the deflection between 0-1 hour and 1-7 hours. The error bars indicate the variability of the variance over the chosen time-step.


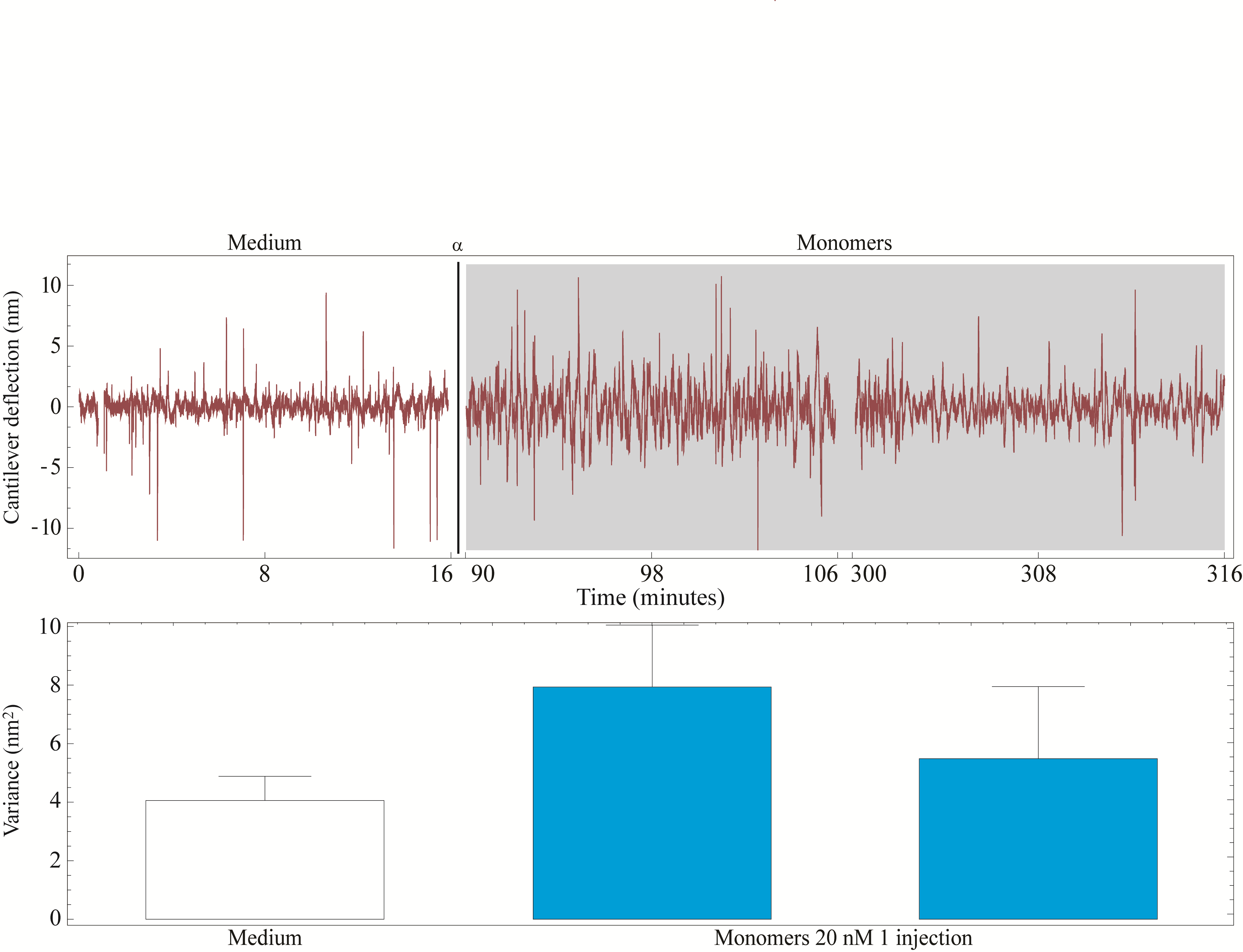

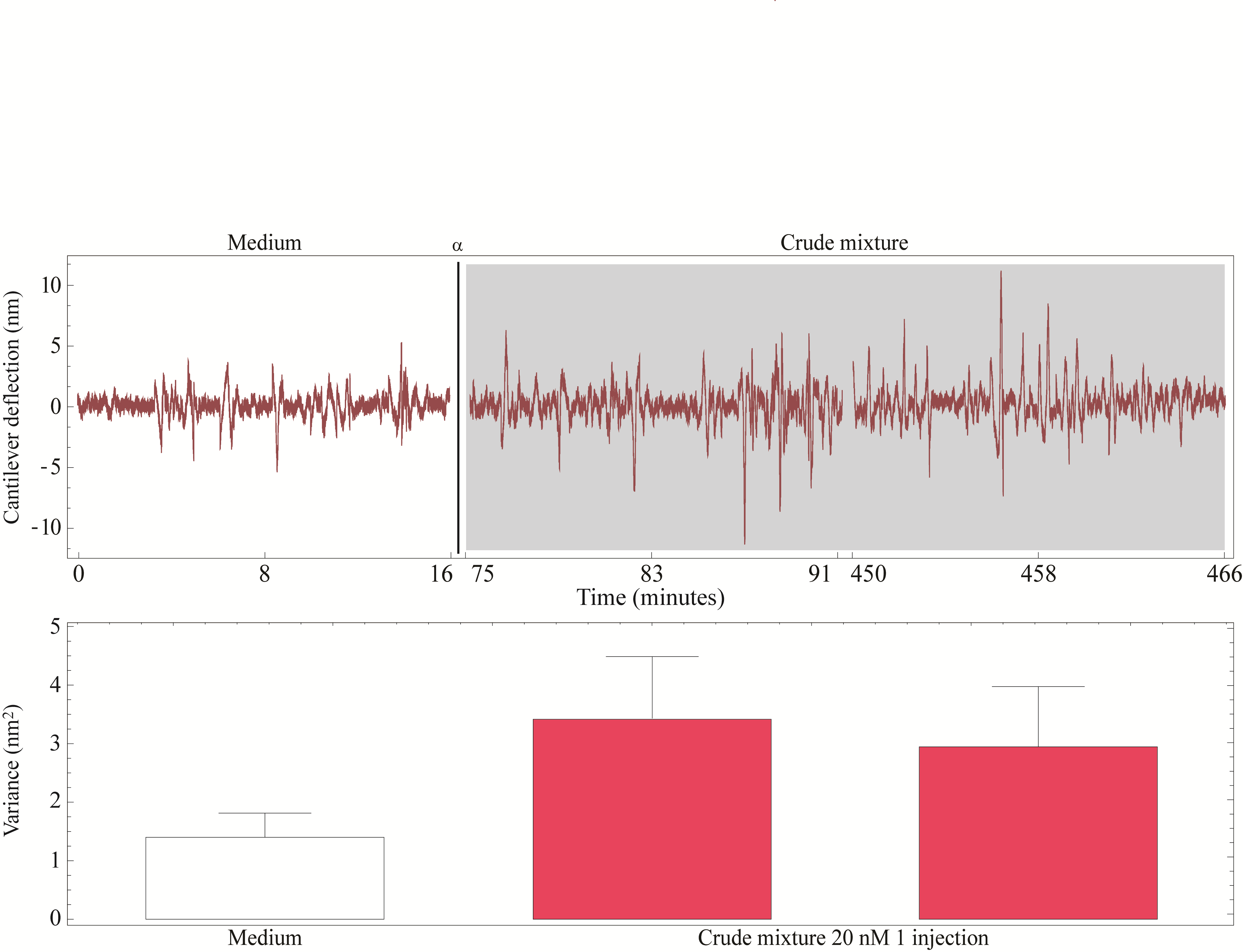

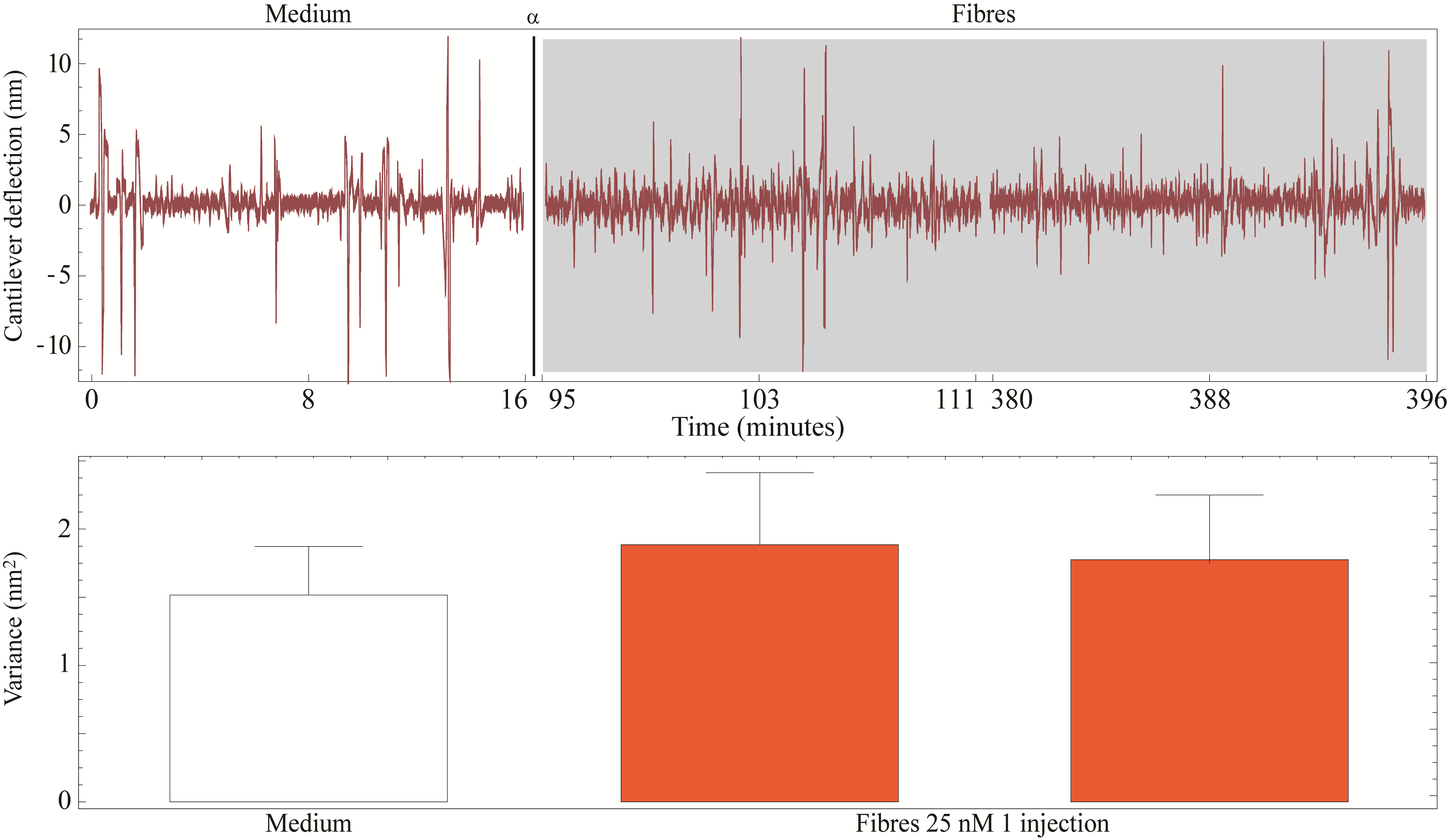


**Figure S4. Nanomotion experiments with monomeric, crude mixture and fibrillar using a single injection.** Typical nanomotion response of M17 neuroblastoma cells exposed to single doses of a) monomeric, b) crude mixture and c) fibrillar α-syn. In all cases, the cells appear viable after 8 hours of monitoring. These results coupled with the fluorescence images in Fig. 4, indicate the localization of the monomer and fibrillar α-syn on the cell membrane. The histograms depict the average variance and the error bars indicate the variability of the variance over the chosen time-step.


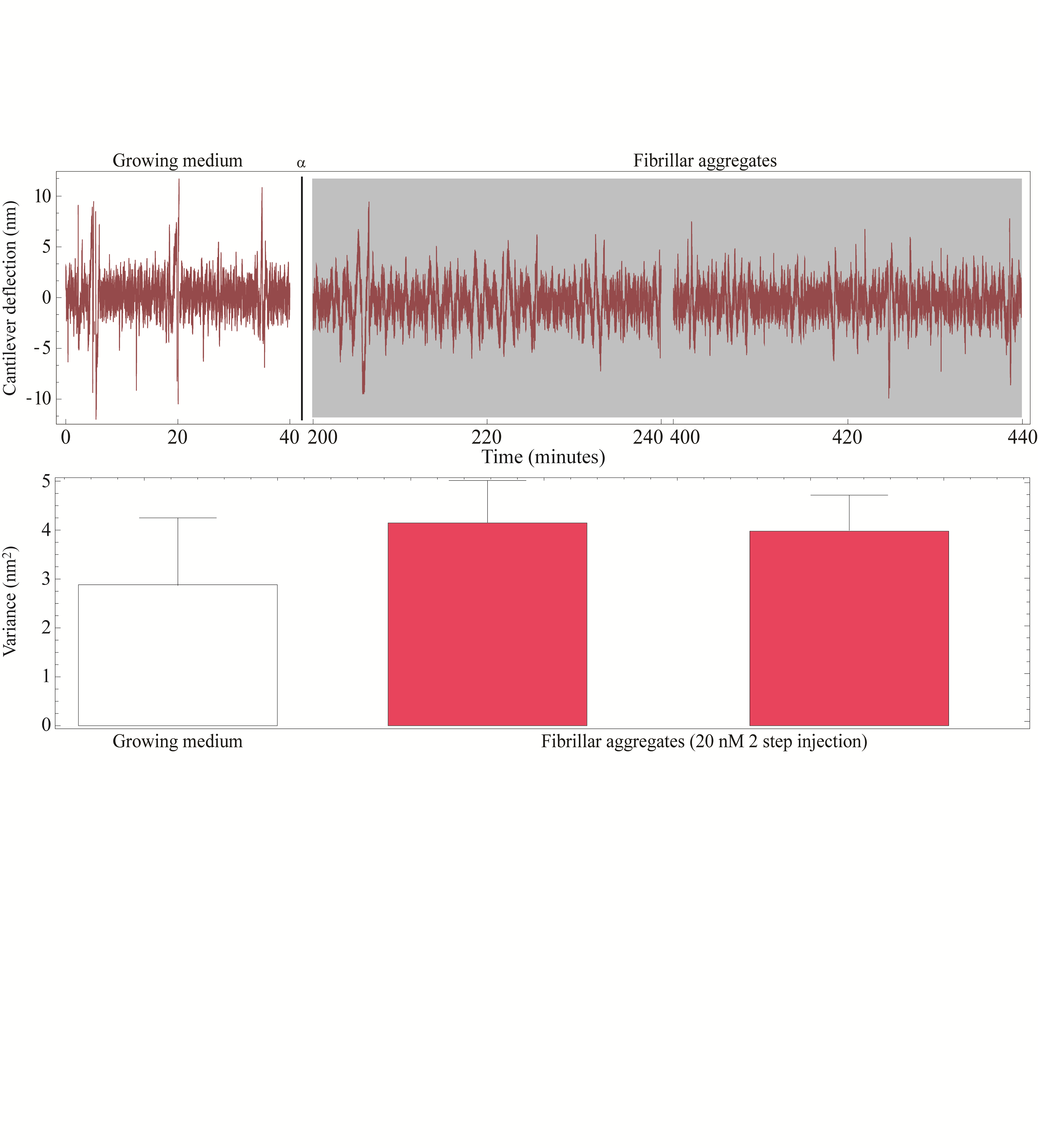


**Figure S5. Nanomotion experiments with pure fibrillar α-syn.** Typical nanomotion response of M17 neuroblastoma cells, out of a triplicate with consistent results, exposed to 2-step fibrillar α-syn. The cells appear viable after several hours of monitoring. The histograms depict the average variance and the error bars indicate the variability of the variance over the chosen time-step.


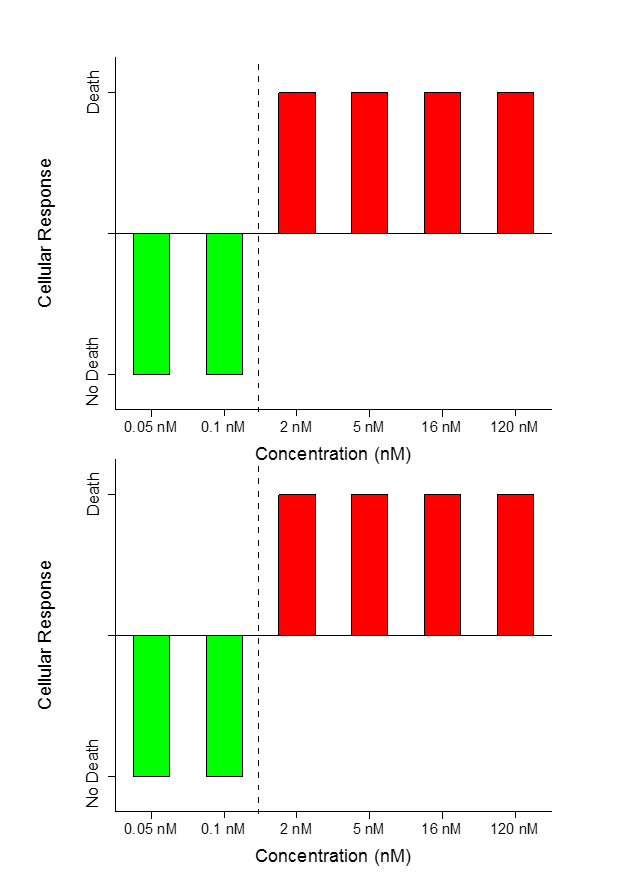


**Figure S6. Cytotoxicity of the crude mixture as a function of injected concentration.** Response of M17 neuroblastoma cells exposed to 2-step crude mixture aggregates. At the concentration of 0.05 nM and 0.1 nM the aggregates do not induce any toxicity, while a minimal concentration of 2 nM is required to cause cellular death within 8 hours after injection.


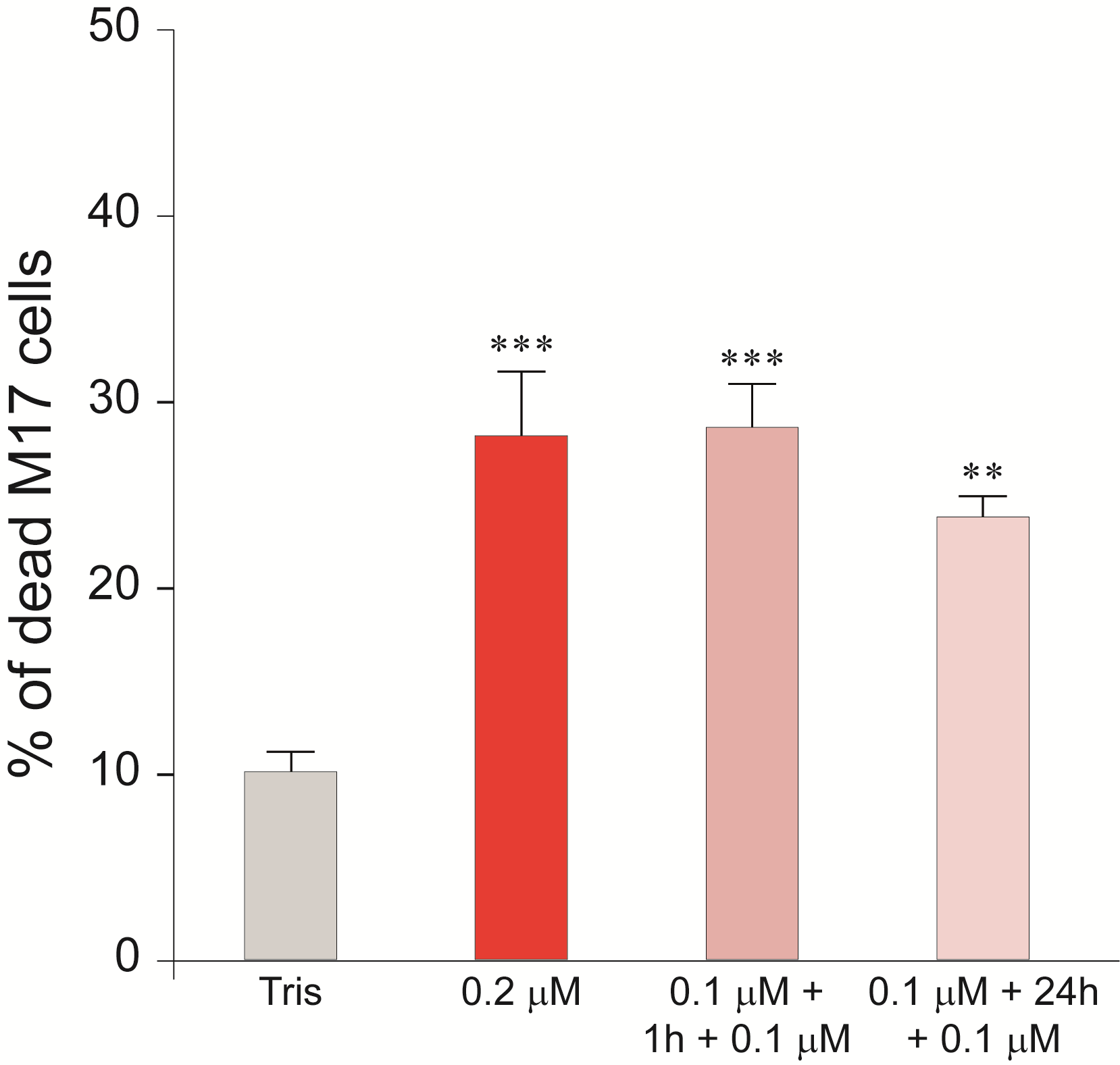


**Figure S7. Cell death quantification by flow cytometry after sequential treatment.** The neuroblastoma M17 cells were treated with Tris buffer (50 mM Tris pH 7.5, 150 mM NaCl; negative control) or with α-syn crude mixture in a single dose or using sequential treatment. The percentage of cell death was measured by flow cytometry. Cell death level is expressed as the percentage of cells with loss of plasma membrane integrity (PI positive cells) to the total cell number analyzed by FACS. Data shown represent the mean of three independent experiments performed in triplicate (bars are mean ± S.D.). One-way ANOVA test followed by a Tukey-Kramer post-hoc test were performed (Tris versus α-syn treated conditions), **p<0.001, ***p<0.0001. No statistical differences were observed between the toxicity induced by α-syn crude mixture added in a single dose or as a sequential treatment.


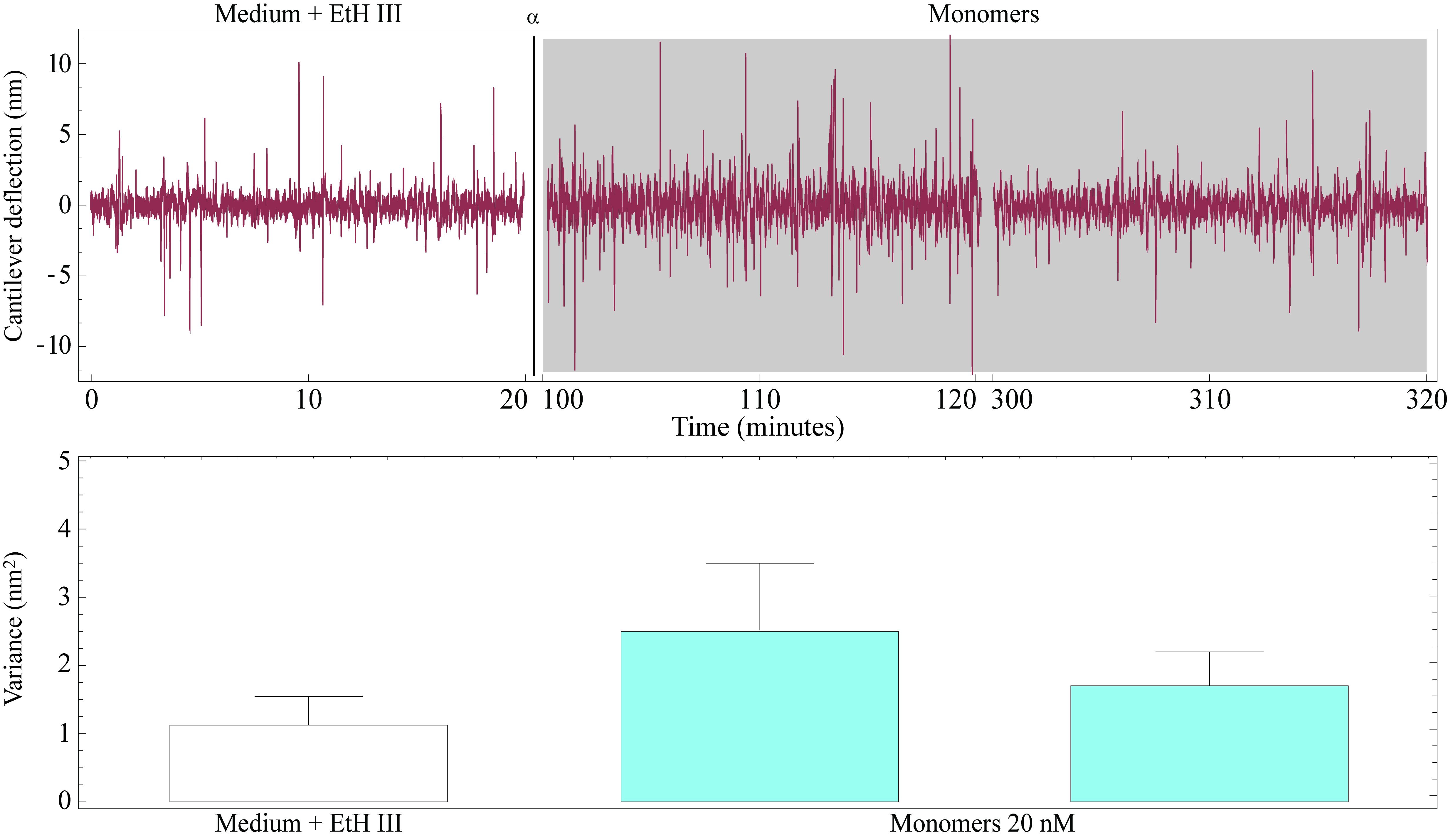


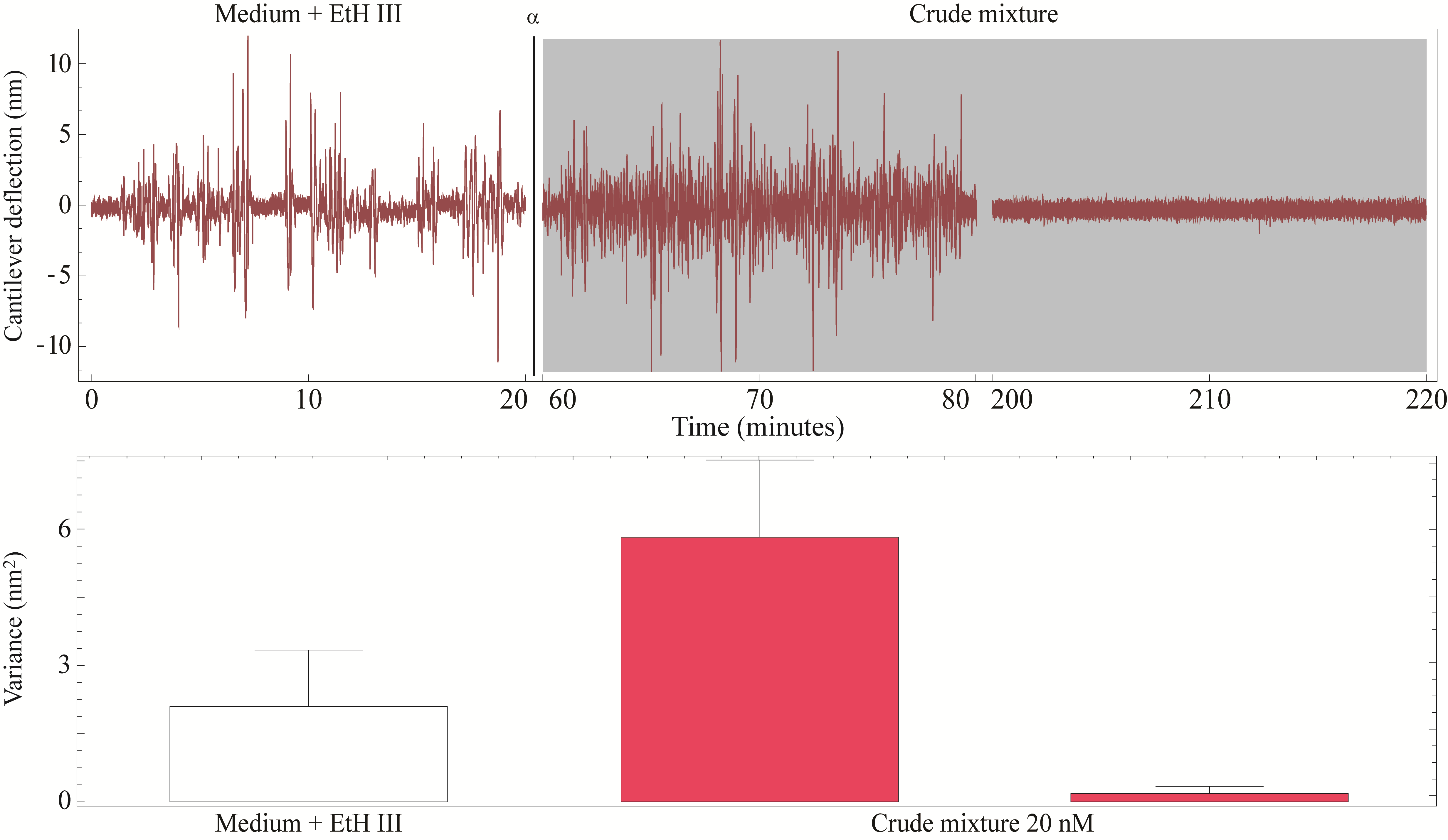


**Figure S8. Nanomotion experiments with double injection in presence of the exclusion vital dye Ethidium homodimer III.** Typical nanomotion response of M17 neuroblastoma cells exposed to EtH III and to monomeric or crude mixture α-syn using the 2-step protocol. The nanomotion results, correlated with the fluorescence images presented in Figure 5, demonstrate the effect of the α-syn on the cell membrane. Each experiment was performed at least 3 times with consistent results. The histograms depict the average variance and the error bars indicate the variability of the variance over the chosen time-step.

**Table S1. Cellular response to sequential α-syn exposure.** Exposure to monomeric and fibrillar α-syn in different order and relative concentrations leading to cell death.

| **1^st^ injection** | **2^nd^ injection** | **Cellular response** |
| --- | --- | --- |
| Monomers (18 nM) | Fibers (2 nM) | Death |
| Fibers (2 nM) | Monomers (18 nM) | Death |
| Fibers (18 nM) | Monomers (2 nM) | Death |
| Monomers (2nM) | Fibers (18 nM) | Death |

**Table S2**. **Time of cellular death after sequential exposure of monomeric and fibrillar α-syn**. Time is calculated as the average of triplicates experiments.

| **1^st^ injection** | **2^nd^ injection** | **Time to death** |
| --- | --- | --- |
| Monomers (18 nM) | Fibrils (2 nM) | 56 ± 4 minutes |
| Fibrils (2 nM) | Monomers (18 nM) | 40 ± 4 minutes |

**Movie captions**

**Movie M1**: Time-lapse video of cells growing on an AFM cantilever in buffer solution. The cells appear to be viable and grow for several hours. The images were collected using a 40x objective. Each frame was collected after 20 seconds and the video was encoded at 20 fps, resulting in each second of the video corresponding to 400 seconds of acquisition.

**Movie M2**: Time-lapse video of cells growing on an AFM cantilever in buffer solution and after the exposure to monomeric α-syn using a 2-step protocol. The cells appear to be viable and grow for several hours. The images were collected using a 40x objective. Each frame was collected after 20 seconds and the video was encoded at 20 fps, resulting in each second of the video corresponding to 400 seconds of acquisition.

**Movie M3**: Time-lapse video of cells growing on an AFM cantilever in buffer solution and after the exposure to the crude mixture of α-syn using a 2-step protocol. After the second injection, the cells appear to rapidly undergo apoptosis.The images were collected using a 40x objective. Each frame was collected after 20 seconds and the video was encoded at 20 fps, resulting in each second of the video corresponding to 400 seconds of acquisition.

Additional References

1 Ruggeri, F. S. *et al.* Influence of the β-Sheet Content on the Mechanical Properties of Aggregates during Amyloid Fibrillization. *Angewandte Chemie* **127**, 2492-2496, (2015).

2 Khalaf, O. *et al.* The H50Q mutation enhances alpha-synuclein aggregation, secretion, and toxicity. *The Journal of biological chemistry* **289**, 21856-21876, (2014).

3 Mahul-Mellier, A. L. *et al.* Fibril growth and seeding capacity play key roles in [alpha]-synuclein-mediated apoptotic cell death. *Cell Death Differ* **22**, 2107-2122, (2015).
